# The Effects of the Combination of a Refined Carbohydrate Diet and Exposure to Hyperoxia in Mice

**DOI:** 10.1155/2016/1014928

**Published:** 2016-11-29

**Authors:** Nicia Pedreira Soares, Keila Karine Duarte Campos, Karina Braga Pena, Ana Carla Balthar Bandeira, André Talvani, Marcelo Eustáquio Silva, Frank Silva Bezerra

**Affiliations:** ^1^Laboratory of Experimental Pathophysiology (LAFEx), Department of Biological Sciences (DECBI), Center of Research in Biological Sciences (NUPEB), Federal University of Ouro Preto (UFOP), Ouro Preto, MG, Brazil; ^2^Laboratory of Metabolic Biochemistry (LBM), Department of Biological Sciences (DECBI), Center of Research in Biological Sciences (NUPEB), Federal University of Ouro Preto (UFOP), Ouro Preto, MG, Brazil; ^3^Laboratory of Immunobiology of Inflammation (LABIIN), Department of Biological Sciences (DECBI), Center of Research in Biological Sciences (NUPEB), Federal University of Ouro Preto (UFOP), Ouro Preto, MG, Brazil; ^4^Laboratory of Experimental Nutrition (LABNEX), School of Nutrition, Ouro Preto, MG, Brazil

## Abstract

Obesity is a multifactorial disease with genetic, social, and environmental influences. This study aims at analyzing the effects of the combination of a refined carbohydrate diet and exposure to hyperoxia on the pulmonary oxidative and inflammatory response in mice. Twenty-four mice were divided into four groups: control group (CG), hyperoxia group (HG), refined carbohydrate diet group (RCDG), and refined carbohydrate diet + hyperoxia group (RCDHG). The experimental diet was composed of 10% sugar, 45% standard diet, and 45% sweet condensed milk. For 24 hours, the HG and RCDHG were exposed to hyperoxia and the CG and RCDG to ambient air. After the exposures were completed, the animals were euthanized, and blood, bronchoalveolar lavage fluid, and lungs were collected for analyses. The HG showed higher levels of interferon-*γ* in adipose tissue as compared to other groups and higher levels of interleukin-10 and tumor necrosis factor-*α* compared to the CG and RCDHG. SOD and CAT activities in the pulmonary parenchyma decreased in the RCDHG as compared to the CG. There was an increase of lipid peroxidation in the HG, RCDG, and RCDHG as compared to the CG. A refined carbohydrate diet combined with hyperoxia promoted inflammation and redox imbalance in adult mice.

## 1. Introduction

Obesity is a public health problem and is correlated with several comorbidities, such as heart failure [1, 2] which, in most cases, requires oxygen supplementation [3]. However, when administering oxygen, professionals should follow a careful method to assess the necessity, time, and dose to be given. Oxygen at high concentrations (hyperoxia) can trigger lung oxidative damage, including damage to components of the extracellular matrix, epithelial and endothelial cell injuries, and lung inflammation [4–6].

According to the World Health Organization, worldwide obesity has doubled since 1980 [7]. In 2005, about 1.6 billion adults over 18 years were overweight, and over 400 million were obese [8]. In 2014, the number of overweight and obese cases increased to more than 1.9 billion and 600 million, respectively [7].

The experimental model of obesity that more closely resembles human obesity is conditioned to foods with high refined carbohydrates and lipids [9]. These macronutrients are responsible for the systemic, chronic low-grade inflammation associated with obesity [10]. Carbohydrates trigger lipogenic enzymes due to the activation of the carbohydrate-responsive element-binding protein (ChREBP), thus favoring the development of obesity [11]. In obesity, the adipocytes release free fatty acids (FFAs) that activate the signaling pathways of inflammation. When FFA binds itself to receptors in the cell membrane of macrophages, it activates a complex of kinase enzymes and protein coding genes involved in the inflammatory response, such as tumor necrosis factor-*α* (TNF-*α*). These proteins activate adipocytes leading to lipolysis that releases more fatty acids and several inflammatory genes [12–14]. TNF-*α* activates the pathway of mitogen-activated protein kinases (MAPKs) responsible for inflammatory gene transcription [15] and can stimulate the infiltration and accumulation of macrophages in adipocytes because of inflammation in obesity [16]. In addition, obesity leads to hypertrophy and hyperplasia of adipocytes, which, in turn, causes hypoperfusion and tissue hypoxia [17, 18]. This process causes a decrease in adiponectin production and an increase in proinflammatory cytokines responsible for inflammation [16, 19].

Obesity and hyperoxia are known to increase reactive oxygen species (ROS) [20, 21]. ROS can be from exogenous or endogenous origins. Endogenous ROS is usually produced as a result of cell metabolism [22, 23]. At low to moderate concentrations, they participate in physiological cellular processes and have a beneficial role in aerobic organisms because of their participation in the regulation of cell signaling, gene expression, and apoptotic mechanisms. However, at high concentrations, ROS may cause damage to cell constituents such as lipids, proteins, and DNA [22]. To counteract ROS, cells have an antioxidant defense system that is either enzymatic or nonenzymatic. Enzymes involved in the primary antioxidant defense system include superoxide dismutase (SOD), catalase (CAT), and glutathione peroxidase [22–24].

Extra care should be taken when administering medicinal oxygen to obese patients, who already have chronic low-grade inflammation [21] and increased ROS [20] and may suffer more severe conditions. Thus, this study aimed to analyze the oxidative and inflammatory effects of a high refined carbohydrate diet in mice exposed to hyperoxia.

## 2. Materials and Methods

### 2.1. Experimental Design

Twenty-four BALB/c mice (male, adults, and 5–7 weeks old) were housed under controlled conditions in standard laboratory cages (Laboratory of Experimental Nutrition, Department of Food, School of Nutrition, Federal University of Ouro Preto) and given free access to water and food. All* in vivo* experimental protocols conducted on the animals at the Federal University of Ouro Preto were approved by the ethics committee (#2013/58). The animals were divided into two groups: the first group (G1) received a standard diet, and the second (G2) received a diet rich in refined carbohydrates, composed of 10% sugar, 45% standard diet, and 45% sweet condensed milk, for twelve weeks. The animal body weight and food intake were measured weekly. After dietary treatment, G1 was randomly divided into the control group (CG) and hyperoxia group (HG), and G2 was randomly divided into the refined carbohydrate diet group (RCDG) and refined carbohydrate diet + hyperoxia group (RCDHG). For 24 hours, the HG and RCDHG were exposed to 100% oxygen, and the CG and RCDG were just exposed to ambient air.

### 2.2. Composition of Diets and Food Intake and Regulation of Body Mass

The animals of the CG and HG were fed standard chow (Labina, Purina; Evialis Group, São Paulo, Brazil), and the RCDG and RCDHG received a high palatability feed, composed of 10% granulated sugar, 45% standard feed, and 45% condensed milk (Nestlé®, São Paulo, Brazil), used to promote obesity in animals [25, 26]. Food intake and body weight gain were measured once a week using a digital scale (Mark®, Series M; Bel Equipment Analytical LTDA, São Paulo, Brazil). To control intake, the diets were weighed before serving to the animals and after a week.

### 2.3. Oral Glucose Tolerance Test (OGTT)

A week before the end of the experiment, the animals were submitted to OGTT, as described by Menezes-Garcia and colleagues [25] and Oliveira and colleagues [25, 26], to investigate their insulin sensitivity.

### 2.4. Exposure to Oxygen

All mice (except the CG and RCDG, which inhaled ambient air) were placed in the inhalation chamber and removed after 24 h. An acrylic inhalation chamber was used to expose the animals to hyperoxia (30 cm long, 20 cm wide, and 15 cm high). Oxygen 100% was purchased from White Martins® (White Martins Praxair Inc., São Paulo, Brazil). The oxygen tank was coupled to the inhalation chamber using a silicone conduit [5, 6, 27]. The oxygen concentration was measured continuously through an oxygen cell (C3, Middlesbrough, England). The mice received water and food* ad libitum*, were kept in individual cages with controlled temperature and humidity (21 ± 2°C, 50 ± 10%, respectively), and were submitted to inverted 12 h cycles of light/dark (artificial lights, 7 p.m. to 7 a.m.).

### 2.5. Euthanasia

After 24 hours of oxygen exposure, all animals were subjected to anesthesia with ketamine (130 mg/kg) and xylazine (0.3 mg/kg) and euthanized by exsanguination. The blood, bronchoalveolar lavage fluid (BALF), and adipose tissues (retroperitoneal, epididymal, and mesenteric) were removed.

### 2.6. Blood Collection

To obtain plasma, two aliquots of blood were collected from each animal in polypropylene tubes containing 15 *µ*L of anticoagulant. One aliquot was sent to the Clinical Analysis Lab Pilot (LAPAC-UFOP) for measurements of blood count and white blood cell count. The other aliquot was centrifuged at 10,000 rpm for 15 min, and the supernatant was removed for cholesterol measurement.

### 2.7. Hemogram and Biochemical Analyses of Blood and Plasma

For the complete blood count, whole blood was diluted with saline (1 : 2), and the erythrocyte hematological parameters, hematocrit and hemoglobin, were evaluated using an electronic counting device (ABX Diagnostics, micro 60, HORIBA®, Tokyo, Japan) at LAPAC-UFOP. Cholesterol concentrations were determined by automatic spectrophotometry using the Random Access Clinical Analyzer (CM-200; Wiener Lab, Rosario, Argentina) and by the enzymatic colorimetric method using a specific kit (Bioclin®; Quibasa, Belo Horizonte, Brazil).

### 2.8. Assessment and Analysis of the BALF

Immediately after euthanasia, the chest of each animal was opened to collect the BALF. The left lung was clamped, the trachea cannulated, and the right lung perfused with 1.5 mL of saline solution. The samples were kept on ice until the end of the procedure to avoid cell lysis. Total, mononuclear, and polymorphonuclear cells were stained with trypan blue, enumerated in a Neubauer chamber (Sigma-Aldrich, MA, USA), and stained again using a fast panoptic coloration kit (Laborclin, Pinhais, Paraná, Brazil) [28, 29]. Differential cell counts were performed on cytospin preparations (Shandon, Waltham, MA, USA) and stained with the fast panoptic coloration kit [30].

### 2.9. Tissue Processing and Homogenization

The right lung was clamped, and a cannula was inserted into the trachea. The airspaces were washed with buffered saline solution (final volume 1.5 mL) maintained on ice. The left lung and epididymal adipose tissue (EAT) were removed and immersed in a fixative solution for 48 hr [6, 30]. The tissue was then processed as follows: tap water bath for 30 min, 70% and 90% alcohol baths for 1 hr each, 2 baths in 100% ethanol for 1 hr each, and embedding in paraffin. For histologic analyses, serial 5 *μ*m sagittal sections were obtained from the left lung and stained with hematoxylin and eosin. The right lung was subsequently homogenized in 1 ml potassium phosphate buffer (pH 7.5) and centrifuged at 1500 ×g for 10 min. The supernatant was collected, and the final volume of all samples was adjusted to 1.5 ml with phosphate buffer. The samples were stored in a freezer (–80°C) for biochemical analyses [30].

## 3. Antioxidant Defense and Oxidative Stress Biomarkers in Lung Homogenates

We used the formation of thiobarbituric acid reactive substances (TBARS) as an index of lipid peroxidation during an acid-heating reaction as previously described by Valenca et al. [31]. Briefly, the TBARS level was estimated in accordance with the method described by Lean et al. [32]. The lung homogenate supernatants (1.0 ml) were mixed with 2.0 ml of TCA-TBA-HCL (15% w/v trichloroacetic acid (TCA); 0.375% w/v thiobarbituric acid (TBA); and 0.25 N hydrochloric acid (HCL)). The solution was heated for 15 min in a boiling water bath. After cooling, the precipitates were removed via centrifugation, and the absorbance of the sample at 535 nm was measured. The TBARS level was calculated using the molar absorption coefficient of malondialdehyde (1.56 × 10^5^ M^−1^ cm^−1.28^).

The lung homogenates were used to determine CAT activity. This method was based on the enzymatic decomposition of hydrogen peroxide (H_2_O_2_) observed spectrophotometrically at 240 nm for 5 min. Ten microliters of the homogenate supernatant was added to a cuvette containing 100 mM phosphate buffer (pH 7.2), and the reaction was initiated by the addition of 10 mM H_2_O_2_. H_2_O_2_ decomposition was calculated using the molar absorption coefficient 39.4 M^−1^ cm^−1^. The results were expressed as activity per mg of protein. One unit of CAT was equivalent to the hydrolysis of 1 *μ*mol of H_2_O_2_ per min [33]. SOD activity was assayed by the spectrophotometric method of Marklund and Marklund [34] using an improved pyrogallol autoxidation inhibition assay. SOD reacts with the superoxide radical (O_2_^−^), and this slows down the rate of formation of o-hydroxy-o-benzoquinone and other polymer products. One unit of SOD is defined as the amount of enzyme that reduces the rate of autoxidation of pyrogallol by 50%.

### 3.1. Adiposity Index

The adipose pads were removed and weighed to determine the adiposity index. The index was calculated by adding the epididymal, retroperitoneal, and mesenteric adipose tissue mass, divided by body weight, and multiplied by 100 [26].

### 3.2. Immunoassays of Epididymal Adipose Tissue (EAT)

The epididymal adipose tissue was used to determine the concentrations of the inflammatory mediators TNF-*α*, IFN-*γ*, and IL-10 and the plasma was used to determine the leptin levels. For the analysis, the samples were thawed and excess proteins were removed by acid/salt precipitation, as previously described [10]. Briefly, equal volumes of epididymal adipose tissue, plasma, and 1.2% trifluoroacetic acid/1.35 M NaCl were mixed, incubated at room temperature for 10 min, and centrifuged for 5 min at 10,000 rpm. The salt content of the supernatant was adjusted to be 0.14 M sodium chloride and 0.01 M sodium phosphate at a pH of 7.4 prior to determination of the concentrations of TNF-*α*, IFN-*γ*, and IL-10 using commercially available ELISA kits (Bio Source International, Inc., CA, USA) and leptin (PeproTech, London, United Kingdom) according to the manufacturer's guidelines. All samples were measured in duplicate [9, 35].

### 3.3. Morphometric and Stereological Analyses

Twenty random images obtained from the histological slides of the lungs and EAT were digitized using a Leica DM5000B optical microscope with Leica Application Suite software and CM300 digital microcamera (Multiuser Laboratory of the Research Center for Biological Sciences of the Federal University of Ouro Preto). The images of the lung and EAT were scanned with 40x and 10x objective lenses, respectively. We used a representative image at 40x magnification with a 100 *µ*m ruler to calibrate a ruler in pixels derived from the program such that 434 pixels equaled 100 *μ*m with the aid of Image J software. Five alveolar areas in each slide prepared from each animal were measured [36, 37]. Six fields from each animal image were captured with a digital camera coupled to a microscope (200x). The area was obtained by randomly measuring 50 adipocytes per blade using the J® Image software (National Institutes of Health, Bethesda, MD, USA).

The analyses of the volume density values of alveolar air space (*V*_*v*_[a]) and volume densities of alveolar septa (*V*_*v*_[sa]) were performed on a test system that consists of sixteen points and a known test area in which the boundary line was considered forbidden in order to avoid overestimation of the number of structures. The test system was matched to a monitor attached to a microscope. The number of points (*P*_*P*_) that touched the alveolar septa was assessed according to the total number of test points (*P*_*T*_) in the system using the equation *V*_*v*_ = *P*_*P*_/*P*_*T*_. To obtain uniform and proportional lung samples, we analyzed 18 random fields in a cycloid test system attached to the monitor screen. The reference volume was estimated by point counting, using the test point system. A total area of 1.94 mm^2^ was analyzed to determine *V*_*v*_sa in slides stained with hematoxylin and eosin [38].

### 3.4. Statistical Analysis

The data with normal distribution were analyzed by unpaired *t*-test, univariate analysis of variance (one-way ANOVA), or by two-way ANOVA followed by Bonferroni's multiple comparison* post hoc* test. Data were expressed as the mean ± standard error of the mean. For discrete data, we used the Kruskal-Wallis test followed by Dunn's* post hoc* test and expressed them as median, minimum, and maximum values. In both cases, the difference was considered significant when the* P* value was less than 0.05. All analyses were performed with GraphPad Prism, version 5.00 for Windows 7 (GraphPad Software; San Diego, CA, USA).

## 4. Results

### 4.1. Food Intake and Body Weight Gain

**Figure 1:**
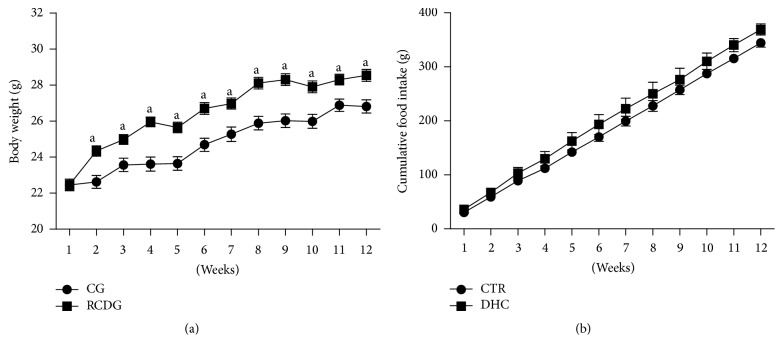
Body weight gain (a) and food intake (b) for 12 weeks. CG: control group; RCDG: refined carbohydrate diet group. Data are presented as mean ± standard error of the mean of six animals per group. ^a^ *P* < 0.05 significantly different values for RCDG and CG. Comparisons were performed using two-way ANOVA followed by Bonferroni's multiple comparison* post hoc* test.

The animals were weighed and their food intake was measured weekly to evaluate if the high refined carbohydrate diet influenced food intake and body weight gain. As shown in Figure 1, the RCDG had a higher body weight gain in the second week of the experiment compared to the CG, and this gain was maintained for 12 weeks. However, no significant difference was observed in the amount (g) consumed by the experimental groups (Figure 1).

### 4.2. Body Adiposity Index, Adipocyte Area, and Leptin

**Figure 2:**
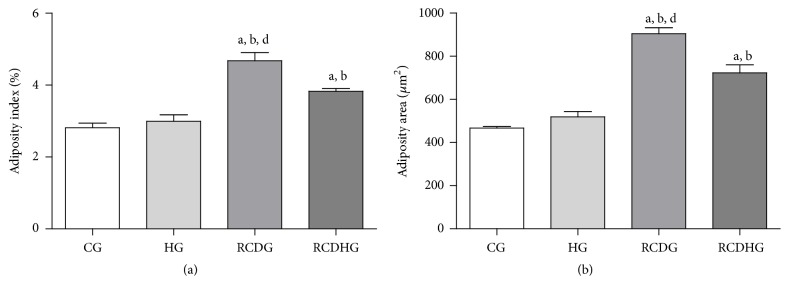
Body adiposity (a) and adipocyte area (b). CG: control group; HG: hyperoxia group; RCDG: refined carbohydrate diet group; RCDHG: refined carbohydrate diet + hyperoxia group. Data are presented as mean ± standard error of the mean of six animals per group. ^a, b, d^ *P* < 0.05 significantly different values for RCDG and CG, HG, and RCHDG, respectively. ^a, b^ *P* < 0.05 significantly different values for RCDHG and CG and HG. Comparisons were performed using one-way ANOVA followed by Bonferroni's multiple comparison* post hoc* test.

**Figure 3:**
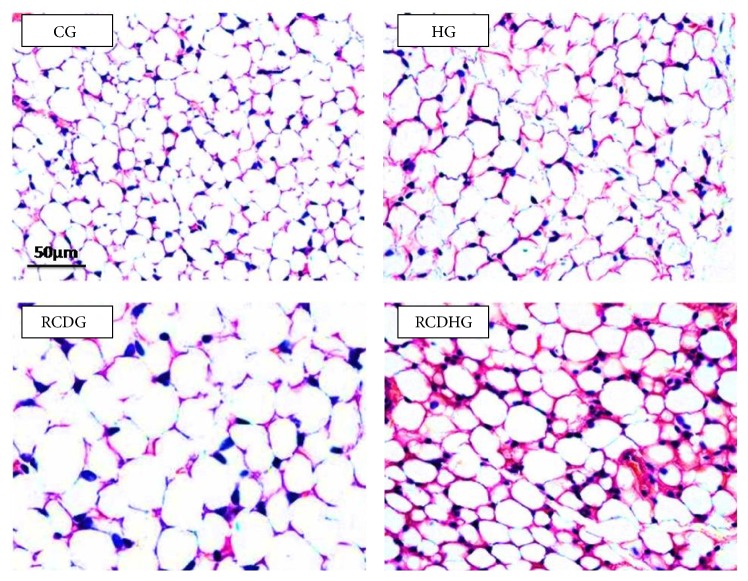
Histological analysis of the epididymal adipose tissue sections stained with hematoxylin and eosin. Bar = 50 *μ*m. CG: control group; HG: hyperoxia group; RCDG: refined carbohydrate diet group; RCDHG: refined carbohydrate diet + hyperoxia group.

**Figure 4:**
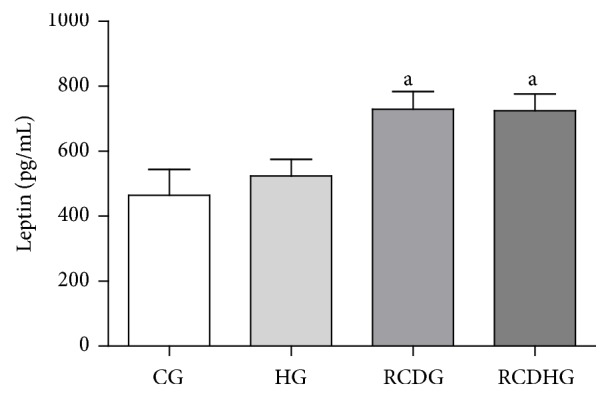
Plasma leptin levels. CG: control group; HG: hyperoxia group; RCDG: refined carbohydrate diet group; RCDHG: refined carbohydrate diet + hyperoxia group. Data are presented as mean ± standard error of the mean of six animals per group. ^a^ *P* < 0.05 significantly different values for RCDG and RCDHG in relation to CG. Comparisons were performed using one-way ANOVA followed by Bonferroni's multiple comparison* post hoc* test.

The diet model induced obesity as confirmed by the body adiposity index, which increased in the RCDG as compared to the other groups (Figure 2), and by the adipocyte area, which increased in the RCDHG compared to the CG and HG, evaluated by morphometric analysis of EAT sections (Figures 2 and 3). There was also an increase of leptin in the RCDG and RCDHG compared to the CG (Figure 4).

### 4.3. Glucose and Cholesterol Metabolism

**Figure 5:**
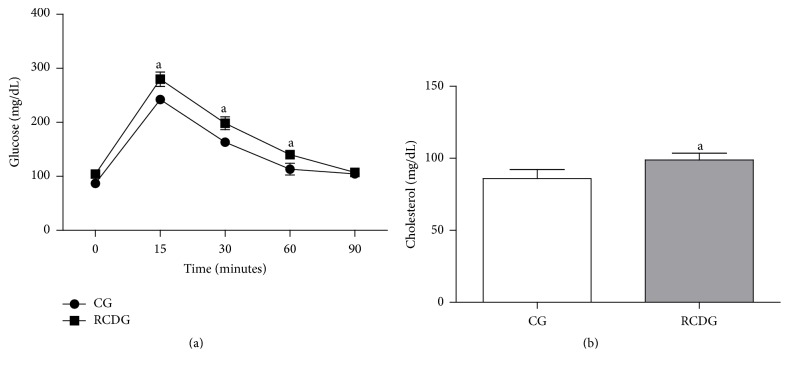
Plasma glucose (a) and cholesterol (b) levels. CG: control group; RCDG: refined carbohydrate diet group. Data are presented as mean ± standard error of the mean six animals per group. ^a^ *P* < 0.05 significantly different values for RCDG compared to the CG. Comparisons were made using two-way ANOVA with Bonferroni's multiple comparison* post hoc* test (a) and unpaired *t*-test (b).

According to the OGTT results, the RCDG presented higher glycemic levels at 15, 30, and 60 min after glucose overload as compared to the CG. There was also a significant increase in total cholesterol in the RCDG showing that this diet was able to induce insulin resistance and hypercholesterolemia in these animals (Figure 5).

### 4.4. Total and Differential Cell Count in the BALF

The dynamics of cell recruitment in the BALF, where the presence of the leukocytes, lymphocytes, neutrophils, and macrophages was identified in the high refined carbohydrate diet and exposure to hyperoxia, was evaluated. As shown in Table 1, there was an increase in the number of total leukocytes in the RCDHG as compared to the CG and HG, as well as an increase in macrophages in the RCDG and RCDHG as compared to the CG and HG.

### 4.5. Total and Differential Blood Cell Count

The total and differential number of cells in the blood was counted. The total leukocytes and lymphocytes in the blood decreased in the HG compared to the CG and RCDG and decreased in the RCDHG compared to the CG (Table 2). Exposure to hyperoxia promoted the significant decrease in neutrophils observed in the HG compared to the CG. Furthermore, the monocytes decreased in the HG and RCDHG compared to the CG.

### 4.6. Stereological Evaluations of Lung Parenchyma of the Experimental Groups

**Figure 6:**
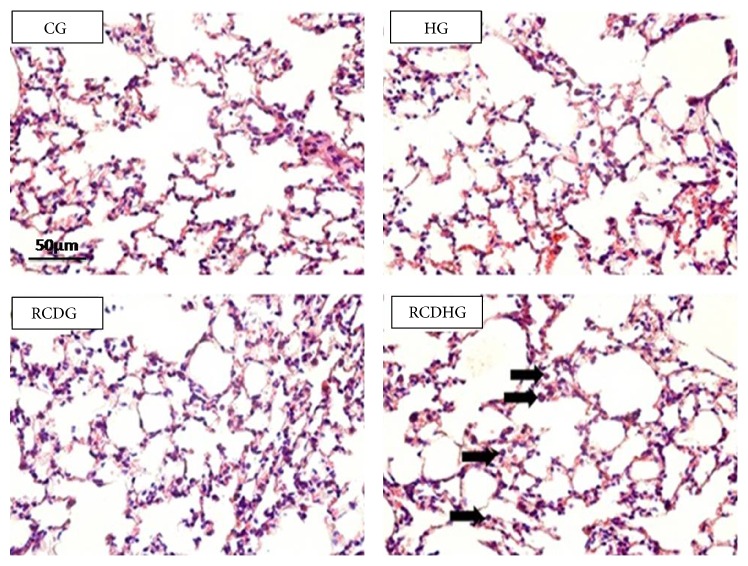
Photomicrographs (400x) of hematoxylin and eosin stained lung sections of the CG, HG, RCDG, and RCDHG. Bar = 50 *μ*m. Cell influx into the lung parenchyma. CG: control group; HG: hyperoxia group; RCDG: refined carbohydrate diet group; RCDHG: refined carbohydrate diet + hyperoxia group. Arrows indicate influx of inflammatory cells into the lung parenchyma in groups exposed to hyperoxia and given a refined carbohydrate diet.

Morphometric analysis showed no significant differences in the alveolar air volume density (*V*_*v*_a) and *V*_*v*_sa in the HG, RCDG, and RCDHG as compared to the CG (Table 3 and Figure 6).

### 4.7. CBC and Biochemical Analysis of the Blood

Clinical hematology is used to evaluate the general state of health of the animal as well as to detect specific diseases [39]. The HG animals had lower serum levels of erythrocytes, hemoglobin, and hematocrit compared to other groups (Table 4).

### 4.8. Immunoenzymatic Assays on the EAT

The immunoenzymatic assays performed on the EAT showed that the HG had higher amounts of IFN-*γ* as compared to the CG, RCDG, and RCDHG and higher levels of IL-10 and TNF-*α* as compared to the CG and RCDHG (Table 5).

### 4.9. Analysis of Redox Imbalance and Damage Caused by Oxidation

The antioxidant enzymes SOD and CAT are generally regulated by oxidative stress and are responsible for the oxidative balance in the lungs. As shown in Table 6, SOD activity in the lung parenchyma decreased in the RCDHG as compared to the CG and HG, and CAT activity decreased in the RCDHG as compared to the CG. The TBARS levels revealed a progressive increase in lipid peroxidation in the HG, RCDG, and RCDHG compared to the CG, as well as in the RCDG compared to the HG and RCDHG (Table 6).

## 5. Discussion

This study showed that a high refined carbohydrate diet increased the mass and body adiposity in experimental animals. This diet has been employed as an obesity induction model in rats and mice and has been accompanied by increased body weight, adiposity, levels of leptin, and plasma concentrations of cholesterol, glucose, and insulin [26, 40]. Our findings showed that a high refined carbohydrate diet resulted in higher resistance to insulin, hypercholesterolemia, and increased levels of leptin in BALB/c mice, corroborating those of Oliveira et al. [26]. Obesity results in several complications in glucose and lipid metabolism, such as the development of insulin resistance, type 2 diabetes, and hyperlipidemia, leading to metabolic syndrome and cardiovascular diseases [21, 38].

Studies have shown that increased adiposity caused by a high palatability diet seems to influence greater food intake [25, 26]; however, there was no influence in food intake on our experimental model. We can speculate the effects of diet composition on metabolic responses since nutrients can act systemically as cellular signals [26]. The higher amount of sucrose in the high refined carbohydrate diet compared to that in the control diet could promote an increase in the activation of lipogenic enzymes due to the activation of ChREBP as well as an increased inflammatory response and insulin resistance [11, 26]. In addition, De Lima et al. (2008) pointed out that high amounts of sucrose in the diet lead to a higher glycemic index, resulting in the elevation of blood glucose and postprandial insulin concentration and favoring fat storage [41]. Therefore, the high concentration of carbohydrates in the high palatability feed possibly contributes to increased body weight, adiposity, adipocyte area, leptin level, glucose intolerance, and hypercholesterolemia.

Studies have shown that both obesity and hyperoxia trigger inflammatory processes [20, 21]. To evaluate whether hyperoxia and/or a high refined carbohydrate diet cause an influx of peripheral blood cells into the lung parenchyma, the total and differential leukocytes present in bronchoalveolar lavage (BAL) and blood of animals were determined. Our results corroborate those of a previous study which reported an increase of macrophages and neutrophils in the BAL of BALB/c mice exposed to hyperoxia for 24 h, though stereological analysis showed no significant difference in *V*_*v*_a among all groups [6]. Our data suggest there was blood cell recruitment to an inflammation site, evidenced by the decrease in the number of leukocytes and lymphocytes in the blood. Our data also showed that hyperoxia might promote inflammation in the adipose tissue of eutrophic mice as evidenced by an increase in the proinflammatory cytokines, IFN-*γ*, and TNF-*α*. Macrophages are the major source of TNF-*α* and other proinflammatory molecules in adipose tissue [42]. Therefore, we suggest that, in addition to the recruitment of macrophages to the lung parenchyma, there was also migration of these cells to adipose tissues. Oliveira et al. (2013) demonstrated that, along with an increase in macrophages in adipose tissues, there is an increase in the number of regulatory T lymphocytes (Tregs), indicating a counterregulatory mechanism to suppress acute inflammation [26]. Since Tregs are directly related to the increase of IL-10 [43], the increase of IL-10 in animals exposed to hyperoxia is justified.

Interferon Gamma (IFN-*γ*) is known to be released after the exposure to hyperoxia by inflammatory cells such as lymphocytes [44]. Our results showed an increase in the levels of IFN-*γ* in adipose tissue in the HG and a decrease of lymphocytes in the blood, indicating possible migration of these cells into the adipose tissue. However, when the animals were subjected to two proinflammatory factors (diet and hyperoxia), there were no significant differences in cytokine levels in adipose tissue, probably for being a secondary tissue injury caused by hyperoxia. Nagato et al. (2012) reported that BALB/c mice exposed to 24 hours of hyperoxia showed an increase in the levels of TNF-*α* and IL-6 in the lung [6]. Furthermore, Naura et al. (2009) showed that animals subjected to a diet High Fat showed an increase in the levels of IFN-*γ* and TNF-*α* in BALF [47]. Thus, we believe that the increase in the levels of cytokines in the lungs of the animals, subjected to hyperoxia and given a refined carbohydrate diet, occurred due to the recruitment of inflammatory cells in the lung without increasing in adipose tissue.

The exposure to hyperoxia promoted a significant decrease of erythrocytes, hemoglobin, and hematocrit in this study. Some studies describe that the low partial oxygen pressure occurs in response to arterial hypoxia by the activation of hypoxia-inducible factor-1 (HIF-1), the main regulator of the hypoxic environment transcription which stimulates the production of erythropoietin, called stimulation-erythrocyte hormone via kidneys acting in the marrow of long bones, stimulating the production of erythrocytes to compensate the low concentration of oxygen in the blood. When the cellular oxygen level is adequate, HIF-1 is degraded [43, 46]. It is possible that there was a decrease in HIF-1 and, consequently, in the production of erythrocytes in animals exposed to hyperoxia.

In this study, the activities of SOD and CAT were evaluated to better understand their contributions to the redox imbalance during exposure to hyperoxia. SOD is major pulmonary defense against the detrimental effects of O_2_ and converts O_2_ to H_2_O_2_, a substrate of CAT. CAT is responsible for preventing H_2_O_2_ accumulation to convert the H_2_O_2_ into two water molecules. The accumulation of H_2_O_2_ possible generates, via Fenton and Haber-Weiss reactions, the hydroxyl radical (^∙^OH) which can react in the side chain and attacks preferably amino acids such as cysteine, histidine, tryptophan, methionine, and phenylalanine, damaging the proteins and, as its consequence, causing the loss of enzyme activity [47]. Diets with high concentrations of lipids and carbohydrates lead to an increase in free fatty acids (FFAs), resulting in an increase of mitochondrial B-oxidation and the overload in the electron transport chain resulting in an increase of production in ROS [39]. Hyperoxia exposes the body to high levels of reactive oxygen species [5, 6] and the high levels of ROS can inhibit the activity of antioxidant enzymes [48]. Thus, the hyperoxia in animals subjected to a high carbohydrate diet causes cell injury, likely by overloading the cellular antioxidant defense, leading to an increase of ROS. In addition, the oxidative load created can reduce levels of antioxidant enzymes and lead to inhibition of their activities [5, 6]. Our results were similar to Nagato et al. (2012) who also reported no significant differences in relation to CAT activity in BALB/C mice exposed to hyperoxia for 24 hours; however, there was a decrease in SOD activity [6]. On the other hand, Nagato et al. (2009) observed a decrease of CAT and SOD in Wistar rats exposed to hyperoxia for 90 minutes [5]. Unlike these previous studies, the animals in this study were exposed to hyperoxia after receiving a high carbohydrate diet for 12 weeks. Thus, we believe that the decrease in the activity of these enzymes was due to the association of these two factors. Besides redox imbalance, hyperoxia causes damage owing to oxidation in the airways, which was supported by studies in BALB/c mice and Wistar rats. This damage can be detected experimentally by monitoring the lipid peroxidation products, such as malondialdehyde [46]. Our results corroborate those of Nagato et al. (2012), who found increased malondialdehyde in BALB/c mice exposed to hyperoxia for 12 and 24 hours [6].

The results of this study, associated with previous studies [5, 6, 44], suggested that the supplemental oxygen is extremely important in clinical practice. However, a special attention should be paid to obese patients who have already had a low intensity chronic inflammation [21] and an increase in ROS [20] which induce lung inflammation [5, 6, 44].

## 6. Conclusions

This study has been the first to report the combined effects of the administration of a high refined carbohydrate diet and the exposure to a high oxygen concentration in adult BALB/c mice. However, more studies should be performed to analyze these effects in other organs or biological systems.

## Figures and Tables

**Table 1:** The inflammatory cells in the bronchoalveolar lavage of experimental groups.

	CG	HG	RCDG	RCDHG	*P*
Leukocytes (×10^3^/mL)	140.0 ± 5.3	146.3 ± 6.0	161.3 ± 5.2	178.3 ± 4.8^a,b^	<0.05
Macrophages (×10^3^/mL)	92.1 ± 9.3	99.6 ± 7.4	128.5 ± 5.0^a,b^	152.9 ± 7.4^a,b^	<0.05
Lymphocytes (×10^3^/mL)	18.2 ± 4.5	9.7 ± 3.5	7.1 ± 2.3	7.6 ± 3.5	>0.05
Neutrophils (×10^3^/mL)	29.7 ± 6.3	37.0 ± 5.1	25.7 ± 5.0	17.9 ± 2.6	>0.05

CG: control group; HG: hyperoxia group; RCDG: refined carbohydrate diet group; RCDHG: refined carbohydrate diet + hyperoxia group. ^a,b^Representing significant differences (*P* < 0.05) compared to CG and HG, respectively. Data were expressed as mean ± SEM (*n* = 6) and were analyzed by one-way ANOVA followed by Bonferroni's multiple comparison *post hoc* test.

**Table 2:** Total and differential leukocyte count in the blood of the experimental groups.

	CG	HG	RCDG	RCDHG	*P*
Leukocytes (×10^3^/mL)	7.2 ± 0.6	2.7 ± 0.4^a,c^	5.4 ± 0.8	4.2 ± 0.5^a^	<0.05
Lymphocytes (×10^3^/mL)	4.9 ± 4.0	1.6 ± 0.3^a,c^	3.6 ± 0.6	2.5 ± 0.3^a^	<0.05
Neutrophils (×10^3^/mL)	1.7 ± 0.1	0.9 ± 0.1^a^	1.3 ± 0.2	1.4 ± 0.2	<0.05
Monocytes (×10^3^/mL)	0.6 ± 0.1	0.2 ± 0.0^a^	0.5 ± 0.1	0.3 ± 0.0^a^	<0.05

CG: control group; HG: hyperoxia group; RCDG: refined carbohydrate diet group; RCDHG: refined carbohydrate diet + hyperoxia group. ^a^Representing significant differences compared to CG. ^a,c^Representing significant differences compared to CG and RCDG, respectively. Data were expressed as mean ± SEM (*n* = 6) and were analyzed by one-way ANOVA followed by Bonferroni's multiple comparison *post hoc* test.

**Table 3:** Comparison of alveolar airspace volume and density of alveolar septa of groups.

	CG	HG	RCDG	RCDHG	*P*
*V*_*v*_a (%)	37.5 (18.7/43.7)	39.1 (25.0/46.8)	39.1 (31.2/46.8)	34.4 (25.0/37.5)	>0.05
*V*_*v*_sa (%)	53.1 (43.7/56.2)	45.3 (43.7/62.5)	43.7 (40.6/50.0)	50.0 (43.7/50.0)	>0.05

*V*_*v*_a: alveolar airspace volume; *V*_*v*_sa: density of alveolar septa. CG: control group; HG: hyperoxia group; RCDG: refined carbohydrate diet group; RCDHG: refined carbohydrate diet + hyperoxia group. Data were expressed as median, minimum, and maximum (*n* = 6) and were analyzed by Kruskal-Wallis test followed by Dunn's *post hoc* test (*P* = 0.95).

**Table 4:** Blood count of the experimental groups.

	CG	HG	RCDG	RCDHG	*P*
RBC (*∗*10^6^/*µ*L)	9.5 ± 0.1	7.6 ± 0.4^a,c,d^	9.3 ± 0.3	9.7 ± 0.2	<0.05
Hemoglobin (g/dL)	17.3 ± 0.3	14.4 ± 0.8^a,c,d^	17.7 ± 0.3	17.6 ± 0.3	<0.05
Hematocrit (%)	52.9 ± 1.0	44.2 ± 2.4^a,c,d^	55.4 ± 1.2	54.4 ± 1.4	<0.05

CG: control group; HG: hyperoxia group; RCDG: refined carbohydrate diet group; RCDHG: refined carbohydrate diet + hyperoxia group. ^a,c,d^Representing significant differences (*P* < 0.05) compared to CG, RCDG, and RCDHG, respectively. Data were expressed as mean ± SEM (*n* = 6) and were analyzed by one-way ANOVA followed by Bonferroni's multiple comparison *post hoc* test.

**Table 5:** Levels of inflammatory markers in epididymal adipose tissue.

	CG	HG	RCDG	RCDHG	*P*
INF-*γ*	549.4 ± 84.8	1027.0 ± 149.4^a,c,d^	558.1 ± 40.7	384.1 ± 88.4	<0.05
IL-10	1.150.0 ± 343.0	2.606.0 ± 568.1^a,d^	1.253.0 ± 166.8	592.5 ± 201.0	<0.05
TNF-*α*	528.0 ± 148.7	1.180.0 ± 245.6^a,d^	608.7 ± 60.7	247.2 ± 79.5	<0.05

CG: control group; HG: hyperoxia group; RCDG: refined carbohydrate diet group; RCDHG: refined carbohydrate diet + hyperoxia group. ^a,c,d^Representing significant differences (*P* < 0.05) compared to CG, RCDG, and RCDHG. ^a,d^Representing significant differences (*P* < 0.05) compared to CG and RCDHG, respectively. Data were expressed as mean ± SEM (*n* = 6) and were analyzed by one-way ANOVA followed by Bonferroni's multiple comparison *post hoc* test.

**Table 6:** Analysis of activities of SOD, CAT, and TBARS in lung samples from the CG, HG, RCDG, and RCDHG groups.

	CG	HG	RCDG	RCDHG	*P*
SOD (U/mg prot)	26.1 ± 1.8	24.4 ± 2.0	20.9 ± 1.7	17.5 ± 1.4^a,b^	<0.05
CAT (U/mg prot)	0.8 ± 0,0	0.7 ± 0.1	0.6 ± 0.1	0.5 ± 0.1^a^	<0.05
TBARS (nM/mg prot)	0.1 ± 0.0	0.3 ± 0.0^a^	0.6 ± 0.0^a,b^	0.8 ± 0.0^a,b,c^	<0.05

CG: control group; HG: hyperoxia group; RCDG: refined carbohydrate diet group; RCDHG: refined carbohydrate diet + hyperoxia group. ^a,b^Representing significant differences (*P* < 0.05) compared to CG and HG. ^a^Representing significant differences (*P* < 0.05) compared to CG. ^a,b,c^Representing significant differences (*P* < 0.05) compared to CG, HG, and RCDG, respectively. Data were expressed as mean ± SEM (*n* = 6) and were analyzed by one-way ANOVA followed by Bonferroni's multiple comparison *post hoc* test.

## References

[1] Rotella C. M., Dicembrini I. (2015). Measurement of body composition as a surrogate evaluation of energy balance in obese patients. *World Journal of Methodology*.

[2] Wong C., Marwick T. H. (2007). Obesity cardiomyopathy: pathogenesis and pathophysiology. *Nature Clinical Practice Cardiovascular Medicine*.

[3] Branson R. D., Johannigman J. A. (2013). Pre-hospital oxygen therapy. *Respiratory Care*.

[4] Nowak D., Kasielski M., Antczak A., Pietras T., Bialasiewicz P. (1999). Increased content of thiobarbituric acid-reactive substances and hydrogen peroxide in the expired breath condensate of patients with stable chronic obstructive pulmonary disease: no significant effect of cigarette smoking. *Respiratory Medicine*.

[5] Nagato A., Silva F. L., Silva A. R. (2009). Hyperoxia-induced lung injury is dose dependent in wistar rats. *Experimental Lung Research*.

[6] Nagato A. C., Bezerra F. S., Lanzetti M. (2012). Time course of inflammation, oxidative stress and tissue damage induced by hyperoxia in mouse lungs. *International Journal of Experimental Pathology*.

[7] WHO (2015). *Obesity and Overweight*.

[8] WHO (2000). *World Health Organization. Obesity: Preventing and Managing the Global Epidemic. Report of a WHO consultation*.

[9] Harrold J. A., Widdowson P. S., Clapham J. C., Williams G. (2000). Individual severity of dietary obesity in unselected Wistar rats: relationship with hyperphagia. *American Journal of Physiology—Endocrinology and Metabolism*.

[10] Bourlier V., Bouloumie A. (2009). Role of macrophage tissue infiltration in obesity and insulin resistance. *Diabetes and Metabolism*.

[11] Benhamed F., Poupeau A., Postic C. (2013). The transcription factor ChREBP: a key modulator of insulin sensitivity?. *Médecine/Sciences*.

[12] Hajer G. R., van Haeften T. W., Visseren F. L. J. (2008). Adipose tissue dysfunction in obesity, diabetes, and vascular diseases. *European Heart Journal*.

[13] Karalis K. P., Giannogonas P., Kodela E., Koutmani Y., Zoumakis M., Teli T. (2009). Mechanisms of obesity and related pathology: linking immune responses to metabolic stress. *The FEBS Journal*.

[14] Arkan M. C., Hevener A. L., Greten F. R. (2005). IKK-*β* links inflammation to obesity-induced insulin resistance. *Nature Medicine*.

[15] Bastos D. H. M., Rogero M. M., Arêas J. A. G. (2009). Effects of dietary bioactive compounds on obesity induced inflammation. *Arquivos Brasileiros de Endocrinologia e Metabologia*.

[16] Tateya S., Tamori Y., Kawaguchi T., Kanda H., Kasuga M. (2010). An increase in the circulating concentration of monocyte chemoattractant protein-1 elicits systemic insulin resistance irrespective of adipose tissue inflammation in mice. *Endocrinology*.

[17] Goossens G. H. (2008). The role of adipose tissue dysfunction in the pathogenesis of obesity-related insulin resistance. *Physiology and Behavior*.

[18] Hosogai N., Fukuhara A., Oshima K. (2007). Adipose tissue hypoxia in obesity and its impact on adipocytokine dysregulation. *Diabetes*.

[19] Wajchenberg B. L., Nery M., Cunha M. R., da Silva M. E. R. (2009). Adipose tissue at the crossroads in the development of the metabolic syndrome, inflammation and atherosclerosis. *Arquivos Brasileiros de Endocrinologia e Metabologia*.

[20] Bonomini F., Rodella L. F., Rezzani R. (2015). Metabolic syndrome, aging and involvement of oxidative stress. *Aging and Disease*.

[21] Chang H.-P., Wang M.-L., Chan M.-H., Chiu Y.-S., Chen Y.-H. (2011). Antiobesity activities of indole-3-carbinol in high-fat-diet-induced obese mice. *Nutrition*.

[22] Bindoli A., Rigobello M. P. (2013). Principles in redox signaling: from chemistry to functional significance. *Antioxidants and Redox Signaling*.

[23] Birben E., Sahiner U. M., Sackesen C., Erzurum S., Kalayci O. (2012). Oxidative stress and antioxidant defense. *World Allergy Organization Journal*.

[24] Cantin A. M., Richter M. V. (2012). Cigarette smoke-induced proteostasis imbalance in obstructive lung diseases. *Current Molecular Medicine*.

[25] Menezes-Garcia Z., Oliveira M. C., Lima R. L. (2014). Lack of platelet-activating factor receptor protects mice against diet-induced adipose inflammation and insulin-resistance despite fat pad expansion. *Obesity*.

[26] Oliveira M. C., Menezes-Garcia Z., Henriques M. C. C. (2013). Acute and sustained inflammation and metabolic dysfunction induced by high refined carbohydrate-containing diet in mice. *Obesity*.

[27] Reis R. B., Nagato A. C., Nardeli C. R., Matias I. C. P., Lima W. G., Bezerra F. S. (2013). Alterations in the pulmonary histoarchitecture of neonatal mice exposed to hyperoxia. *Jornal de Pediatria*.

[28] Silva Bezerra F., Valença S. S., Lanzetti M. (2006). *α*-Tocopherol and ascorbic acid supplementation reduced acute lung inflammatory response by cigarette smoke in mouse. *Nutrition*.

[29] Lanzetti M., Bezerra F. S., Romana-Souza B. (2008). Mate tea reduced acute lung inflammation in mice exposed to cigarette smoke. *Nutrition*.

[30] Oliveira T. H. V. D., Campos K. K. D., Soares N. P., Pena K. B., Lima W. G., Bezerra F. S. (2015). Influence of sexual dimorphism on pulmonary inflammatory response in adult mice exposed to chloroform. *International Journal of Toxicology*.

[31] Valenca S. S., Castro P., Pimenta W. A. (2006). Light cigarette smoke-induced emphysema and NF*κ*B activation in mouse lung. *International Journal of Experimental Pathology*.

[32] Lean M. E. J., Han T. S., Deurenberg P. (1996). Predicting body composition by densitometry from simple anthropometric measurements. *American Journal of Clinical Nutrition*.

[33] Buege J. A., Aust S. D. (1978). Microsomal lipid peroxidation. *Methods in Enzymology C*.

[34] Aebi H. (1984). Catalase in vitro. *Methods in Enzymology*.

[35] Silva R. R., Shrestha-Bajracharya D., Almeida-Leite C. M., Leite R., Bahia M. T., Talvani A. (2012). Short-term therapy with simvastatin reduces inflammatory mediators and heart inflammation during the acute phase of experimental Chagas disease. *Memórias do Instituto Oswaldo Cruz*.

[36] Hauner H. (2004). The new concept of adipose tissue function. *Physiology and Behavior*.

[37] Esposito K., Pontillo A., Giugliano F. (2003). Association of low interleukin-10 levels with the metabolic syndrome in obese women. *Journal of Clinical Endocrinology and Metabolism*.

[38] Treacher D. F., Leach R. M. (1998). ABC of oxygen: oxygen transport—1. Basic principles. *British Medical Journal*.

[39] Nagato A. C., Bezerra F. S., Talvani A., Aarestrup B. J., Aarestrup F. M. (2015). Hyperoxia promotes polarization of the immune response in ovalbumin‐induced airway inflammation, leading to a TH_17_ cell phenotype. *Immunity, Inflammation and Disease*.

[40] Imai K., D'Armiento J. (2000). Activation of an embryonic gene product in pulmonary emphysema: identification of the secreted frizzled-related protein. *CHEST Journal*.

[41] De Lima D. C., Silveira S. A., Haibara A. S., Coimbra C. C. (2008). The enhanced hyperglycemic response to hemorrhage hypotension in obese rats is related to an impaired baroreflex. *Metabolic Brain Disease*.

[42] Marinou K., Tousoulis D., Antonopoulos A. S., Stefanadi E., Stefanadis C. (2010). Obesity and cardiovascular disease: from pathophysiology to risk stratification. *International Journal of Cardiology*.

[43] Hao Q., Lillefosse H. H., Fjære E. (2012). High-glycemic index carbohydrates abrogate the antiobesity effect of fish oil in mice. *American Journal of Physiology—Endocrinology and Metabolism*.

[44] Weisberg S. P., McCann D., Desai M., Rosenbaum M., Leibel R. L., Ferrante A. W. (2003). Obesity is associated with macrophage accumulation in adipose tissue. *The Journal of Clinical Investigation*.

[45] Feuerer M., Herrero L., Cipolletta D. (2009). Lean, but not obese, fat is enriched for a unique population of regulatory T cells that affect metabolic parameters. *Nature Medicine*.

[46] Valença S. D. S., Kloss M. L., Bezerra F. S., Lanzetti M., Silva F. L., Porto L. C. (2007). Effects of hyperoxia on Wistar rat lungs. *Jornal Brasileiro de Pneumologia*.

[47] Naura A. S., Hans C. P., Zerfaoui M. (2009). High-fat diet induces lung remodeling in ApoE-deficient mice: an association with an increase in circulatory and lung inflammatory factors. *Laboratory Investigation*.

[48] Berger P., Karpel Vel Leitner N., Doré M., Legube B. (1999). Ozone and hydroxyl radicals induced oxidation of glycine. *Water Research*.

